# Pressure and Volume Limited Ventilation for the Ventilatory Management of Patients with Acute Lung Injury: A Systematic Review and Meta-Analysis

**DOI:** 10.1371/journal.pone.0014623

**Published:** 2011-01-28

**Authors:** Karen E. A. Burns, Neill K. J. Adhikari, Arthur S. Slutsky, Gordon H. Guyatt, Jesus Villar, Haibo Zhang, Qi Zhou, Deborah J. Cook, Thomas E. Stewart, Maureen O. Meade

**Affiliations:** 1 Interdepartmental Division of Critical Care, University of Toronto, Toronto, Canada; 2 Keenan Research Centre and Li Ka Shing Knowledge Institute, St. Michael's Hospital, Toronto, Canada; 3 Department of Critical Care Medicine and Sunnybrook Research Institute, Sunnybrook Health Sciences Centre, Toronto, Canada; 4 Department of Clinical Epidemiology and Biostatistics, McMaster University, Hamilton, Canada; 5 CIBER de Enfermedades Respiratorias, Instituto de Salud Carlos III, Madrid, Spain; 6 Research Unit, Hospital Universitario Dr. Negrin, Las Palmas de Gran Canaria, Spain; Oregon Health and Science University, United States of America

## Abstract

**Background:**

Acute lung injury (ALI) and acute respiratory distress syndrome (ARDS) are life threatening clinical conditions seen in critically ill patients with diverse underlying illnesses. Lung injury may be perpetuated by ventilation strategies that do not limit lung volumes and airway pressures. We conducted a systematic review and meta-analysis of randomized controlled trials (RCTs) comparing pressure and volume-limited (PVL) ventilation strategies with more traditional mechanical ventilation in adults with ALI and ARDS.

**Methods and Findings:**

We searched Medline, EMBASE, HEALTHSTAR and CENTRAL, related articles on PubMed™, conference proceedings and bibliographies of identified articles for randomized trials comparing PVL ventilation with traditional approaches to ventilation in critically ill adults with ALI and ARDS. Two reviewers independently selected trials, assessed trial quality, and abstracted data. We identified ten trials (n = 1,749) meeting study inclusion criteria. Tidal volumes achieved in control groups were at the lower end of the traditional range of 10–15 mL/kg. We found a clinically important but borderline statistically significant reduction in hospital mortality with PVL [relative risk (RR) 0.84; 95% CI 0.70, 1.00; p = 0.05]. This reduction in risk was attenuated (RR 0.90; 95% CI 0.74, 1.09, p = 0.27) in a sensitivity analysis which excluded 2 trials that combined PVL with open-lung strategies and stopped early for benefit. We found no effect of PVL on barotrauma; however, use of paralytic agents increased significantly with PVL (RR 1.37; 95% CI, 1.04, 1.82; p = 0.03).

**Conclusions:**

This systematic review suggests that PVL strategies for mechanical ventilation in ALI and ARDS reduce mortality and are associated with increased use of paralytic agents.

## Introduction

Acute lung injury (ALI) and its most severe form, acute respiratory distress syndrome (ARDS), are common life-threatening complications of critical illness. While support with mechanical ventilation is crucial for survival, use of ventilators without regard for lung volumes and airway pressures may perpetuate lung injury and contribute to the associated high mortality of these clinical conditions. Despite recent randomized controlled trials (RCTs), the benefit of current ventilation strategies designed to limit iatrogenic lung injury remains controversial.

In 1964, Greenfield et al proposed that mechanical ventilation can induce lung injury. [1] Subsequent laboratory investigations established a direct relationship between exposure to increasing tidal volumes and airway pressures, and the development of pulmonary lesions identical to those that characterize ARDS. [2], [3] These findings are consistent across species and in various models of ARDS. [4] One proposed mechanism of injury includes selective over-distention of the diminished volume of functional lung tissue in ARDS. [5] Supporting these preclinical findings, early clinical observations suggested that ventilation strategies to reduce tidal volumes and airway pressures could improve survival. [6]–[8].

These observations challenged the conventional primary goal of mechanical ventilation, which was to achieve normal arterial blood gas values. Accordingly, clinicians used tidal volumes in the range of 10–15 mL/kg with no particular restrictions of airway pressures. [9] In 1993, a Consensus Conference of experts sponsored by the *American College of Chest Physicians* recommended that plateau airway pressures should not exceed 35 cm H_2_O and tidal volumes could be reduced to 5 mL/kg or less to achieve this pressure threshold, even if hypercapnia ensued [10]. The most notable physiological effect of this approach is respiratory acidosis, which can be associated with air hunger, agitation, and patient-ventilator asynchrony, [11] hemodynamic compromise, and acute kidney injury, although evidence for the latter effects is limited. [12].

Several RCTs and meta-analyses [13]–[16] exploring the role for pressure and volume-limited (PVL) ventilation strategies in ALI and ARDS diverged in their conclusions. One systematic review of 6 trials involving 1297 patients concluded that PVL reduces mortality at 28 days and at hospital discharge. [16] In contrast, an analysis of 5 trials involving 1,202 patients concluded that “low tidal volumes should not be standard for these patients.” [13] Additional trials have been published since these reports. Our objective was to systematically review all RCTs comparing PVL to more traditional ventilation strategies for adults with ALI and ARDS to clarify the effects on mortality and other relevant outcomes, and to explore differences among study results.

## Methods

We conducted this review according to current standards for systematic review and meta-analysis, [17] using a predefined protocol.

### Search Strategy

We electronically searched Medline (1966-July 2010), EMBASE (1980-July 2010), HEALTHSTAR (1975-July 2010), and CENTRAL (to July 2010) without language restrictions, and hand-searched abstracts published in the *American Journal of Respiratory and Critical Care Medicine*, *Chest*, *Intensive Care Medicine* and *Critical Care Medicine* (1995–2006). We also screened the reference lists, searched the related articles feature on PubMed™, and contacted investigators on each trial selected for review.

### Trial Selection

Reviewers (KB, NA, MM) independently screened all titles and abstracts in duplicate (except conference proceedings) and then the full articles of all potentially relevant citations. We selected RCTs including critically ill patients, of which at least 80% were adults, at least 80% were mechanically ventilated, and at least 80% had ALI (using author's definitions). We resolved disagreements by consensus.

Conceptually, we were interested in trials comparing ventilation strategies that differed with respect to tidal volumes, airway pressures, or both. Therefore, in addition to trials comparing ventilation strategies with explicit constraints on tidal volumes or airway pressures, we also considered trials that observed an incidental gradient in tidal volume (at least 3 mL/kg) or plateau pressure (at least 5 cm H_2_O) during the first 7 days of study. We included trials that reported on mortality, barotrauma, duration of mechanical ventilation, use of sedation or paralytic agents, need for acute dialysis, or non-pulmonary organ dysfunction. We excluded quasi-randomized trials, such as those assigning patients by alternate allocation or hospital file number, and trials evaluating high frequency ventilation or oscillation, extracorporeal circulation, or implantable devices to augment gas exchange.

### Data Abstraction

Two reviewers (KB, MM) independently abstracted data and methodological features, resolving disagreements in consultation with a third reviewer. We contacted trial investigators for relevant unpublished data and to obtain trial databases. Two reviewers (KB, MM) worked together to collate data with the assistance of a data analyst.

### Validity Assessment

We assessed: allocation concealment, baseline similarity of groups (with regard to age, severity of illness, severity of lung injury, airway pressures, non-pulmonary organ dysfunction, and duration of hospitalization); relevant cointerventions (management of acidosis, application of positive end-expiratory pressure [PEEP], prone positioning, inhaled nitric oxide, systemic corticosteroids, sedation and weaning protocols), and early stopping. [18] We used the GRADE approach to summarize the quality of evidence for each outcome. [19] In this approach, randomized trials begin as high quality evidence but can be rated down for apparent risk of bias, imprecision, inconsistency, indirectness, or suspicion of publication bias.

### Quantitative Data Synthesis

To assess effects of PVL on hospital mortality, we used the most protracted follow up in each trial up to hospital discharge. We explored as potential effect modifiers: i) incorporation of ‘open lung’ techniques (using authors' definitions) into experimental PVL strategies; ii) varied thresholds for correcting respiratory acidosis; iii) between-group gradients in tidal volumes, and airway pressures; and iv) case mix effects. We reasoned that each of these might influence the effect of PVL on mortality. To explore a modifying influence of ‘open lung’ strategies, we compared pooled effects among studies with and without ‘open lung’ strategies.

To assess tolerance for respiratory acidosis we planned 4 separate analyses and resolved to report positive findings only if results were consistent. Two subgroup analyses assessed the effect of different approaches to acidosis management. Trials were classified by their pH thresholds for sodium bicarbonate administration as either above or below (i) a clinically reasonable pH threshold (7.25), and (ii) the mean pH threshold across trials. In addition, we conducted 2 univariate meta-regressions [20] to assess the impact on mortality of (i) the pH threshold at which the assigned tidal volume or airway pressure could deviate from protocol and (ii) mean pH thresholds across trials on mortality. For trials that did not allow protocol deviations at pH extremes, we assigned a pH level of 7.00.

To assess the influence of between-group gradients in tidal volume, we conducted meta-regressions of the gradients in assigned tidal volumes, and meta-regressions of the gradients in tidal volumes achieved on day 1. We used the same approach to assess the influence of variable airway pressure gradients and the impact of having mean airway pressures in the 2 groups spanning a threshold of 30 cm H_2_O. We hypothesized that treatment effects would be greater in trials in which mean day 1 airway pressures (ideally plateau airway pressures, if available) in the 2 groups were on either side of 30 cm H_2_O.

To explore the influence of case mix, we evaluated 2 baseline variables in separate meta-regressions: mean age and mean baseline arterial partial pressure of oxygen/fractional concentration of inspired oxygen ratio (PaO_2_/FiO_2_). Baseline data on plateau airway pressure were insufficiently reported to evaluate this variable as an effect modifier. Data on the Lung Injury Score (LIS) [21] and the Acute Physiology and Chronic Health Evaluation (APACHE) II Score [22] were too inconsistently reported to evaluate as effect modifiers.

To assess the effect of PVL on barotrauma we pooled trial estimates of the relative risk of barotrauma, using authors' definitions. We also explored the influence of ‘open lung’ ventilation, gradients in assigned and achieved (day1) tidal volume, and airway pressure gradients. To assess the influence of PVL on acute dialysis, we pooled study estimates of the relative risk of instituting dialysis.

We planned to evaluate the effects of PVL on duration of mechanical ventilation and ICU and hospital length of stay. Recognizing that early deaths systematically underestimate the duration of these outcomes among survivors, we pooled data for these outcomes separately among survivors and non-survivors. We also planned sensitivity analyses to explore the influence of weaning and sedation protocols on these outcomes.

We used random effects models [23] to pool results from each trial (taking into consideration variation within and between trials) and Review Manager 4.2.8 software (Cochrane Collaboration, Oxford) to derive summary estimates of relative risk (RR) with 95% confidence intervals (CI) for binary outcomes. We formally assessed for heterogeneity using the Cochran Q statistic [23] (with a threshold p-value <0.10 [24]) and the I^2^ measure. [25], [26] I^2^ values of 0–40%, 30–60%, 50–90% and ≥75% represented modest, moderate, substantial, and considerable heterogeneity, respectively [17]. In exploring possible explanations for heterogeneity, we formally assessed for between-group differences in summary estimates using the z-test for interaction. We tested for publication bias using funnel plot analysis. We conducted meta-regressions using SAS version 9.1 (SAS Institute, Cary, NC).

## Results

### Flow of Included Studies

**Figure 1:**
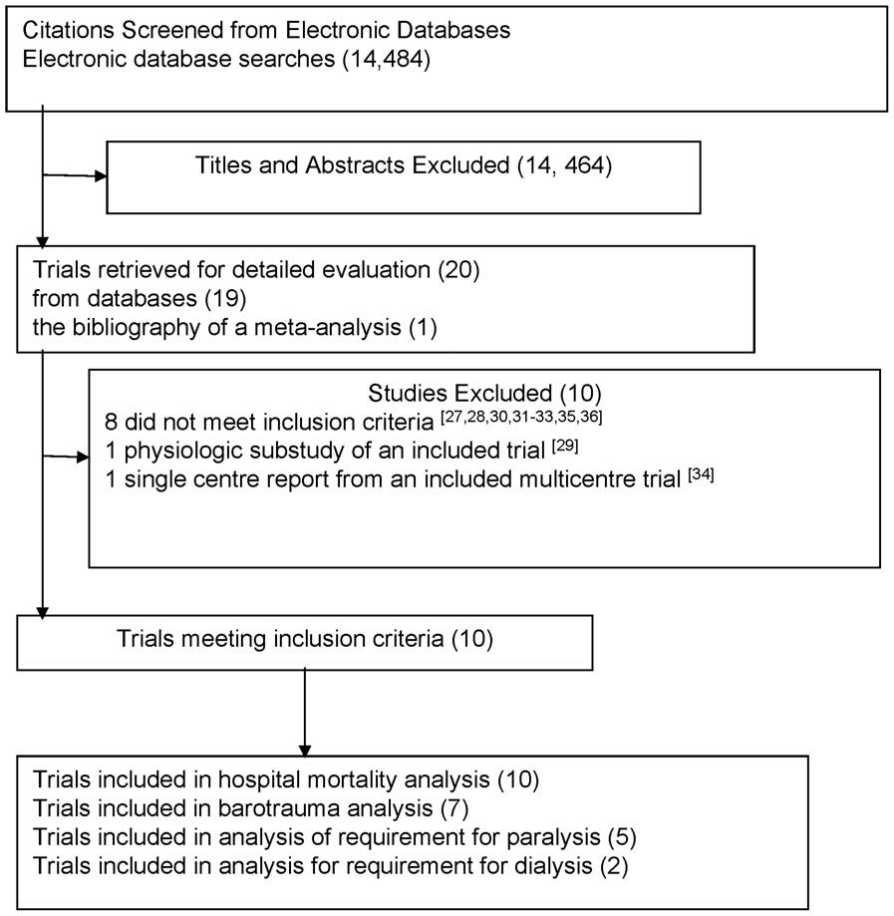
Trials Evaluated During the Systematic Review of the Literature.

After evaluation of 14,484 citations, 20 references were evaluated in detail, and 10 were excluded. [27]–[36] Ten trials [37]–[46] involving 1,749 patients met selection criteria (Figure 1). Reviewers achieved complete agreement on trial selection and reasons for exclusion (Table 1). For 2 trials published both in full [39], [42] and in part [34], [35] both sources informed this review. We had access to 2 complete trial databases [40], [43] and 3 partial databases. [38], [39], [41] We contacted several trial investigators [37], [39]–[46] to clarify study procedures. The lead investigator of a foreign language publication [37] of 56 patients, not reported as randomized, confirmed that the trial was randomized. [37].

**Table 1:** Table of Excluded Studies.

Study[year]	Reason for Exclusion
Lee^[27]^[1990]	Included a small number of patients with ALI (14.6%).
Rappaport^[28]^[1994]	Neither compared the desired alternative approaches to ventilation nor achieved a gradient in tidal volume or airway pressure during follow-up
Carvalho^[29]^[1997]	Physiologic substudy of an included trial.^[38]^
Ranieri^[30]^[1999]	Randomized trial implemented over a 40 hour study period.
Esteban^[31]^[2000]	Neither compared the desired alternative approaches to ventilation or achieved a gradient in tidal volume or airway pressure during follow-up.
Niu^[32]^[2000]	Neither compared the desired alternative approaches to ventilation or achieved a gradient in tidal volume or airway pressure during follow-up.
Long^[33]^[2006]	Neither compared the desired alternative approaches to ventilation or achieved a gradient in tidal volume or airway pressure during follow-up
McKinley^[34]^[2001]	Single centre substudy of an included larger, multicentre trial^[41]^.
Amato^[35]^[1995]	Preliminary data from an included trial^[38]^.
Wang^[36]^[2007]	Did not achieve difference between treatment groups in plateau airway pressure or tidal volumes.

### Study Characteristics

Table 2 summarizes trial protocols. [37]–[46] Two trials [42], [46] were not explicitly designed to compare PVL with more traditional strategies, but noted an incidental difference in mean tidal volume and plateau airway pressures between groups and consequently met criteria for inclusion. One trial [42] was designed to compare a computer-based versus paper-based implementation of the same PVL protocol, and the investigators observed a reduction in both tidal volumes and airway pressures in the experimental group. The other trial [46] compared low-stretch ventilation guided by peak or alternatively plateau airway pressures, depending on the ventilator mode utilized, to low tidal volume ventilation. In 2 trials, the experimental PVL strategy (and not the control group strategy) incorporated liberal PEEP [39] and recruitment maneuvers. [45] In another trial [46] a liberal PEEP chart and recruitment maneuvers were permitted in both treatment arms.

**Table 2:** Study Populations and Protocols.

StudyYear[Sample Size]	InclusionCriteria	VentilatorModes	PVL Strategy		Control Strategy		PEEP^a^	pH Thresholds^c^
			Tidal Volume	Airway Pressure	Tidal Volume	Airway Pressure		
Wu^[37]^1998[56]	PaO_2_/FiO_2_ <300,PaO_2_ <60 mm Hg, Infiltrates,Risk factor for ARDS	AC and SIMV/PS	7–10 cc/kgDry BW**		10–15 cc/kgDry BW**		Suggested guidelines Titrated to PaO_2_Range: 3–12 cm H_2_O	
Brochard^[38]^1998[116]	LIS >2.5 for <72 h Bilateral infiltratesSingle organ failure	AC	6–10 cc/kgActual BW¶	P_PLAT_ ≤25 cm H_2_Oor ≤30 cm H_2_O if FiO_2_≥0.90, reduced chest wall compliance or pH <7.05	10–15 cc/kgActual BW¶	PIP ≤60 cm H_2_O	Explicit protocolPre study PEEP trialNo titration during studyRange: 0–15 cm H_2_O	pH <7.05- violate V_T_,- NaHCO3,^−^- dialysis
Amato^[39]^1998[53]	LIS ≥2.5Risk factor for ARDS	PS, PCIRV or volume ensured PS (PVL)PCIRV if FiO_2_≥0.50 (PVL) AC or controlled ventilation (Control)	<6 cc/kgActual BW	PIP <40 cm H_2_ODriving pressure (Pplat – PEEP) <20 cm H_2_O	12 cc/kgActual BW		Explicit protocolsPVL: 2 cm H_2_O aboveLIP (recruitment maneuvres) Control: Titrated to Fi0_2_	pH <7.20- NaHCO3^−^
Stewart^[40]^1998[120]	PaO_2_/FiO_2_ <250 at PEEP 5 cm H_2_ORisk factor for ARDS	ACPC if PIP consistently at threshold	≤8 cc/kgIdeal BW‡	PIP ≤30 cm H_2_O	10–15 cc/kgIdeal BW‡	PIP ≤50 cm H_2_O	Suggested guidelines Titrated to FiO_2_ and PaO_2_ Range: 5–20+^b^ cm H_2_O	pH <7.0- NaHCO3^−^- violate PIP,- if refractoryprotocol violations at MD discretion
Brower^[41]^1999[52]	PaO_2_/FiO_2_ ≤200 Bilateral infiltrates	AC and SIMV/PS(≤5 cm H_2_O)	5–8 cc/kg Predicted BW§	P_PLAT_ <30 cm H_2_O	10–12 cc/kg Predicted BW§	P_PLAT_ <45–55 cm H_2_O	Explicit protocolTitrated to FiO_2_ and PaO_2_ Range: 5–20+^b^ cm H_2_O	pH <7.20- Adjust RR (max 30 b/min)- pH <7.30 NaHCO3^−^permitted andrequired if pH <7.20
*East^[42]^1999[200]	PaO_2_/FiO_2_≤200 Bilateral infiltrates Risk factor for ARDS Static compliance≤50 ml/cm H_2_O	AC (PVL)	6 cc/kg(6–10 cc/kg)Ideal BW Computerized protocol	Airway pressure not controlled	Clinician discretion	NA	PVL: Explicit protocol Titrated to FiO_2_ and PaO_2_ Range: 5–25 cm H_2_0 Control: Clinician discretion	Target pH = 7.30(range: 7.25 –7.35)- V_T_ (PVL) range- 6–10 cc/kg- RR (PVL) max 35 b/min
ARDS Network^[43]^2000[861]	PaO_2_/FiO_2_≤300Bilateral infiltrates	AC	6 cc/kg(4–8 cc/kg) Predicted BW§	P_PLAT_≤30 cm H_2_O	12 cc/kgPredicted BW§	P_PLAT_≤50 cm H_2_O	Explicit protocolTitrated to FiO_2_ and PaO_2_ Range: 5–24 cm H_2_O	If 7.15≤ pH ≤7.30RR to max 35 or pH>7.30 or PaCO2 <25If RR = 35 or PaCO2 <25 may give NaHCO3^−^If pH <7.15RR to max 35, If RR = 35 or PaCO_2_ <25 NaHCO3^−^, violate V_T_ by 1cc/kg and exceed P_Plat_
McKinley^[34]^2001[67]	PaO_2_/FiO_2_≤200 Bilateral infiltrates Risk factor for ARDSStatic compliance<50 ml/cm H_2_O	AC (PVL)	6 cc/kg(6–10 cc/kg) Ideal BW Computerized protocol	Airway pressure not controlled	Clinician discretion	PIP <50 cm H_2_O: procedure manual	PVL: explicit protocol Titrated to FiO_2_ and PaO_2_ Range: 5–25 cm H_2_O Control: Suggested guideline and clinician discretion	Target pH = 7.30(range: 7.25 –7.35)- V_T_ (PVL) range- 6–10 cc/kg- RR (PVL) max 35 b/min
Orme^[44]^2003[120]	PaO_2_/FiO_2_≤150 Infiltrates in at least 3 of 4 quadrantsRisk factor for ARDS	AC	4–8 cc/kgPredicted BW†	P_PLAT_ <40 cm H_2_O	10–15 cc/kgPredicted BW†	P_PLAT_ <70 cm H_2_O	Computerized protocols or rules (both groups) Titrated to PaO_2_>55 mm Hg adjusting FiO_2_ and PEEP	If pH <7.35 (HTV) orpH <7.20 (LTV)- Adjust RR,- dialysis,- NaHCO3^−^
Villar^[45]^2006[103]	PaO_2_/FiO_2_≤200 on PEEP 5, VT 5 cc/kg ×24 hrsBilateral infiltrates Criteria persist ×24 h	ACP-AC if barotrauma	5–8 cc/kgPredicted BW§	PIP <35–40 cm H_2_O	9–11 cc/kgPredicted BW§	PIP <35–40 cm H_2_O	PVL: 2 cm H_2_O above LIP or 13 cm H_2_OControl: PEEP ≥5 cm H_2_O	pH: clinician discretion PaCO_2_ between 35–50 mm Hg
Sun^[46]^2009[85]	PaO_2_/FiO_2_≤300 Bilateral infiltrates PAWP ≤18 mm Hg	V-AC (PVL)P-AC or SIMV+PS or PS (Control)	4–6 cc/kgPredicted BW	P_PLAT_≤30 cm H_2_O	Target: ∼12 cc/kg PBW	PIP ≤35 cm H_2_O (P-AC) or P_PLAT_ ≤30 cm (PS)	Explicit protocol [42] Titrated to FiO_2_ and PaO_2_ Range: 5–24 cm H_2_O	If pH <7.20 receivedNaHCO3^−^ until pH >7.30

*Details obtained from a separate publication of a subgroup with trauma-induced ARDS by McKinley et al (n = 67)^[34]^.

PVL = pressure and volume-limitation, LIS = lung injury score^[21]^, PEEP = positive end expiratory pressure, AC = volume cycled, P-AC = pressure assist control, V-AC = volume assist control, AC = assist control mode, PC = pressure control mode, PCIRV = pressure control inverse ratio ventilation, SIMV = synchronized intermittent mandatory ventilation, PS = pressure support, BW = body weight, P_PLAT_ = plateau airway pressure, PIP = peak inspiratory pressure, LIP = lower inflection point, PaO_2_ = arterial partial pressure of arterial oxygen, PaCO_2_ =  partial pressure of arterial carbon dioxide, SpO_2_ = oxygen saturation by pulse oximetry, FiO_2_ = fractional concentration of oxygen in inspired gas, NaHCO3^−^ = sodium bicarbonate, RR = respiratory rate, IRV = inverse ratio ventilation, V_T_ = tidal volume, HTV  =  high tidal volume, LTV = low tidal volume, PAWP  =  pulmonary artery wedge pressure.

**Formulae:**

** Dry BW: Actual body weight minus the estimated weight gain due to salt and water retention.

§ Predicted body weight (PBW): male PBW  = 50+2.3 [height (inches) –60]; female PBW  = 45.5+2.3 [height (inches) – 60]. Alternatively, male PBW  = 50+0.91 (centimeters of height –152.4); female PBW  = 45.5+0.91 (centimeters of height –152.4).

† PBW based on actuarial data.

‡ IBW (kg) =  height (meters)^2^×25.

¶ Actual Body Weight minus the estimated weight gain due to water and salt retention.

**Assessment:**

a PEEP: Line 1: We assessed for the presence of an explicit protocol, suggested guideline or titration of PEEP at physician discretion; Line 2: description of initial settings or titration to specific parameters; Line 3: details the range of PEEP permitted.

b PEEP >20 cm H_2_O permitted if profoundly hypoxemic.

c pH Thresholds: We assessed for a threshold pH value (or pH range) and strategies utilized to increase pH.

Table 3 summarizes study methods, highlighting features related to the risk of bias. Randomization was concealed in all trials (unclear in 2) and follow up was excellent. Limitations included lack of central randomization (3 trials), and incomplete reporting of potentially relevant co-interventions (5 trials). Additional threats included the necessary lack of blinding (all trials) and stopping early for benefit (3 trials) or futility (3 trials).

**Table 3:** Scientific Quality of Experimental Methods.

Study[Year]	Allocation Concealment	Baseline Similarity^a^	Experimental Cointerventions^b^	Sedation^c^	Weaning^d^	Early Stopping^e^
**Wu** ^[**37**]^ **[1998]**	Sealed envelopes	Not specified	None	Clinician discretion	Clinician discretion	Yes,Futility
**Brochard** ^[**38**]^ **1998]**	Sealed envelopes	Age: similarPulmonary injury: similar(PaO_2_/FiO_2_, LIS)Illness severity: similar (APACHE II^[22]^, SAPS II^[47]^)	Nitric oxideFrequent but similar	Clinician discretion	Clinician discretion	Yes,Futility,*a priori* rules
**Amato** ^[**39**]^ **[1998]**	Sealed, opaque, sequentially numbered envelopes	Age: similarPulmonary injury: modestly favors controls(PaO_2_/FiO_2_, LIS^[21]^, Static compliance)Illness severity: similar (APACHE II^[22]^, CCS^[48]^, organ failures)Sepsis: favors controls	Recruitment maneuvresPVL group onlyNo others	Suggested guideline (sedation type; not amount)	Suggested guideline	Yes,Benefit,*a priori* rules
**Stewart** ^[**40**]^ **[1998]**	Sealed, opaque sequentially numbered envelopes	Age: similarPulmonary injury: modest favors controls (PaO_2_/FiO_2_, oxygen index)Illness severity: similar (APACHE II^[22]^, MODS^[49]^)Sepsis: favors controls	No nitric oxideNo prone positioning	Clinician discretion	Clinician discretion	No
**Brower** ^[**41**]^ **[1999]**	Independent randomization centre	Age: similarPulmonary injury: modestly favors controls (PaO_2_/FiO_2_, LIS^[21]^)Illness severity: similar (APACHE III^[50]^)Sepsis: similar	No nitric oxideProne position: NA	Clinician discretion	Clinician discretion	Yes,Futility,*a priori* rules
* **East** ^[**42**]^ **[1999]**	Independent randomization centre	NA	None	Clinician discretion	PVL: explicit protocol Control: clinician discretion	No
**ARDS Network** ^[**43**]^ **[2000]**	Independent randomization centre	Age: similarPulmonary injury: similar (PaO_2_/FiO_2_, ARDS)Illness severity: similar (APACHE III^[50]^, organ failure)Sepsis: similar	Prone positionRare but similarOthers: <1%	Clinician discretion	Explicit protocol	Yes,Benefit,a priori rules
**McKinley** ^[**34**]^ **[2001]**	Independent randomization centre	Age: similarIllness severity: similar (ISS^[51]^)	None (PVL) group	Suggested guideline and clinician discretion	PVL: explicit protocol Control: clinician discretion	No
**Orme** ^[**44**]^ **[2003]**	Sealed, opaque, sequentially numbered envelopes	NA	Unknown	Suggested guideline	Explicit protocol	No
**Villar** ^[**45**]^ **[2006]**	Sealed, opaque, envelopes	Age: similarPulmonary injury: similar (LIS^[21]^)Illness severity: similar (APACHE II^[22]^)Sepsis: similar	NA	Clinician discretion	Clinician discretion	Yes,Benefita priori rules
**Sun** ^[**46**]^ **[2009]**	Assigned numbers, Random number table	Age: similarPulmonary injury: similar proportion with PaO_2_/FiO_2_<200 mm HgIllness severity: similar(APACHE II^[22]^)Sepsis: similar	No steroids or, inhaled Nitric oxide (both groups) Prone position occasionally at MD discretionRecruitment maneuvers (both groups)	Suggested guideline (type, amount, route) Protocolized daily awakening	Explicit Protocol (both groups)	No

* Details obtained from a separate publication of a subgroup with trauma-induced ARDS by McKinley et al (n = 67)^[34]^.

PVL = pressure and volume-limitation; LIS = lung injury score^[21]^; APACHE II  =  Acute Physiology and Chronic Health Evaluation II^[22]^; SAPS  =  simplified acute physiology score^[47]^; CCS = clinical classification score^[48]^; MODS =  multiple organ dysfunction score^[49]^; APACHE III  =  Acute Physiology and Chronic Health Evaluation^[50]^; ISS = illness severity score^[51]^; NA = not available.

**Assessment:**

a Baseline similarity: Factors assessed at the time of randomization included Line 1: *age*; Line 2: *severity of pulmonary injury (*PaO_2_/FiO_2_ or LIS^[21]^ or A-a gradient or oxygen index or compliance); Line 3: *illness severity* (APACHE II^[22]^ or SAPS^[47]^ or MODS^[49]^ or APACHE III^[50]^ or ISS^[51]^ and Line 4: *sepsis.* Each variable was assessed as similar between treatment groups or favoring PVL or control.

b Experimental cointerventions: Line 1: We assessed for the use of corticosteroids, inhaled nitric oxide, prone positioning, high frequency oscillation, extracorporeal circulation and surfactant; Line 2: We described the frequency with which experimental cointerventions were utilized in each study and between treatment groups within studies.

c Sedation: We characterized sedation management as guided by an explicit protocol, suggested guideline or at the discretion of clinicians.

d Weaning: We characterized weaning management as guided by an explicit protocol, suggested guideline or at the discretion of clinicians.

e Early stopping: Line 1: We assessed for early trial termination; Line 2: We assessed whether the study stopped early for benefit or futility and Line 3We assessed for explicit *a priori* stopping rules.

Table 4 summarizes the evolution of respiratory variables in the course of each trial, depicting actual between-groups gradients in tidal volumes and airway pressures. Along with Table 2, this table highlights features relevant to the generalizability of this review, including the limits of tidal volumes and airway pressures in the control groups. Reported mean tidal volumes in control and pressure and volume limited groups ranged from 9.8 to 12 mL/kg and 6.1 to 9.0 mL/kg, respectively, while the between group gradient in achieved tidal volume ranged from 2.0 to 5.6 mL/kg over the first week of study.

**Table 4:** Study Implementation.

StudyYear[N]	PopulationAge,PaO_2_/FiO_2_LIS^[21]^,APACHE^[22]^	PEEPTimePEEP Gradient Achieved (cm H_2_O)PEEP Achieved - Control Group (cm H_2_O)PEEP Achieved - PVL Group (cm H_2_O)				Tidal VolumeTimeTidal Volume Gradient Achieved (cc/kg or cc)Tidal Volume - Control Group (cc/kg or cc)Tidal Volume - PVL Group (cc/kg or cc)				Airway PressureTimeAirway Pressure Gradient Achieved (cm H_2_O)Airway Pressure - Control Group (cm H_2_O)Airway Pressure - PVL Group (cm H_2_O)			
Wu^[37]^1998[56]	40.3 ± 12.7———												Unspecified time 9.9 cm H_2_O∥ 24.1 14.2
Brochard^[38]^1998[116]	56.8 ± 15.3149.5 ± 64.63.0 ± 0.317.5 ± 7.5	Day 1010.710.7	Day 20.210.810.6	Day 7−1.18.59.6	Day 140.58.37.8	Day 13.2 cc/kg†10.37.1	Day 23.43 cc/kg†10.57.07	Day 73.33 cc/kg†10.77.37	Day 142.3 cc/kg†9.97.6	Day 16.0 cm H_2_O†31.725.7	Day 25.9 cm H_2_O†31.325.4	Day 76.0 cm H_2_O‡30.524.5	Day 149.1 cm H_2_O‡33.624.5
Amato^[39]^1998[53]	34.4 ± 13.5122.0 ± 58.83.3 ± 0.427.5 ± 6.6	36 hours−7.7†8.716.4	Days 2-7−3.9∥9.313.2			36 hours420 cc¶†768348	Days 2 – 7351 cc¶ †738387			36 hours6.7 cm H_2_O†36.830.1	Days 2 – 713.9 cm H_2_O†37.823.9		
Stewart^[40]^1998[120]	58.5 ± 18.0134.0 ± 60.8—22.0 ± 8.5	Day 1−1.4∥7.28.6	Day 3−0.38.48.7	Day 7−1.68.09.6		Day 13.7 cc/kg†10.77.0	Day 33.6 cc/kg†10.87.2	Day 73.3 cc/kg†10.16.8		Day 14.5 cm H_2_O‡26.822.3	Day 36.3 cm H_2_O‡28.522.2	Day 78.6 cm H_2_O‡28.620.0	
Brower^[41]^1999[52]	48.4 ± 15.8139.5 ± 60.72.8 ± 0.587.6§ ± 26.8					Days 1 - 52.9 cc/kg†10.27.3				Days 1 - 55.7 cm H_2_O†30.624.9			
ARDS Network^[43]^2000[861]	51.5 ± 17.5136.0 ± 61.1—82.5§ ± 28.0	Day 1−0.8∥8.69.4	Day 3−0.6∥8.69.2	Day 71.0∥9.18.1		Day 15.6 cc/kg∥11.86.2	Day 35.6 cc/kg∥11.86.2	Day 74.9 cc/kg∥11.46.5		Day 18.0 cm H_2_O∥33.025.0	Day 38.0 cm H_2_O∥34.026.0	Day 711.0 cm H_2_O∥37.026.0	
*McKinley^[34]^2001[67]	39.0 ± 2.5———	Day 1−1.010.011.0	Day 3010.010.0	Day 5010.010.0		Day 12.011.09.0	Day 34.012.08.0	Day 53.011.08.0		Day 11.038.037.0	Day 3 4.0 39.0 35.0	Day 55.040.035.0	
Villar^[45]^2006[103]	49.9—2.9±0.4518.0 ± 6.5	Day 1−5.1†9.014.1	Day 3−2.5∥8.711.2	Day 60.18.38.2		Day 12.9†10.27.3	Day 32.9†10.07.1	Day 62.8†9.97.1		Day 12.032.630.6	Day 34.1∥32.528.4	Day 66.7†32.425.7	
Sun^[46]^2009[85]	50.5 ± 13.2——83.5 ± 28.0							1^st^ week3.79.86.1				1^st^ week1.025.726.7	

* Details obtained from a separate publication of East et al^[42]^ including a subgroup with trauma-induced ARDS by McKinley et al (n = 67)^[34]^.

PVL = pressure and volume-limitation,

* Subgroup of a multicentre RCT comparing protocol directed, pressure and volume-limited ventilation to non-protocol directed ventilation by East et al (n = 200)^[42]^.

Gradients reflect the difference between the control and treatment groups (i.e. Control – Treatment).

Characteristics of the study populations are presented as pooled mean and standard deviation.

¶ idal volume in cc.

§ APACHE III.

**Significance Levels**.

† p≤0.001;

‡ 0.001<p<0.01;

∥ 0.01≤p≤0.05.

### Quantitative Data Synthesis

#### Mortality

**Figure 2:**
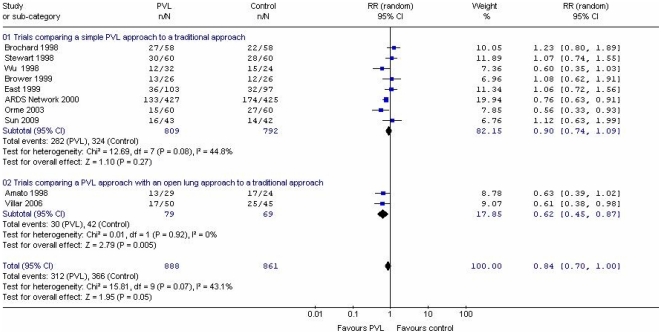
Forest Plot of Hospital Mortality.

One trial measured “death before discharge home and breathing without assistance” and reported a statistically significant difference favoring a PVL approach (RR 0.78; 95%CI 0.65, 0.93). [43] To derive hospital mortality data from this trial, we used individual patient data. Figure 2 illustrates individual trial estimates of the relative risk of hospital mortality, which varied across trials in both magnitude and direction. In 10 trials [37]–[46] involving 1,749 patients we found lower hospital mortality with PVL (RR 0.84; 95% CI 0.70, 1.00, p = 0.05), with moderate heterogeneity (I^2^ = 43.1%, p = 0.07). (Figure 2) In a sensitivity analysis excluding 2 trials [39], [45] that used ‘open lung’ strategies (0.62; 95% CI 0.45, 0.87, p = 0.005), the finding of improved hospital mortality with PVL was attenuated (RR 0.90; 95% CI 0.74, 1.09, p = 0.27), and results could not rule out the possibility of important benefit or harm with PVL. In a *post hoc* sensitivity analysis, excluding a single trial [37] stated by the authors to be randomized but not using this descriptor in the publication, the RR of hospital mortality using PVL was 0.86 (95% CI 0.72, 1.04; p = 0.11).

The various analyses that we conducted to assess tolerance for acidosis with PVL strategies as an effect modifier generated inconsistent results and were, therefore, inconclusive (data not shown). Meta-regression analyses did not identify the magnitude of within-study gradients in assigned (or achieved) tidal volumes or airway pressures between treatment groups as important effect modifiers (data not shown). We did not find a linear relationship between study mean age or mean baseline PaO_2_/FiO_2_ and mortality. However, these analyses were underpowered and limited by the small number of included studies (range 3 to 9).

#### Barotrauma

Rates of barotrauma varied across trials from 3.8% [41] to 41.7%. [39] Pooling across 7 trials [38]–[43], [45] including 1,497 patients, the relative risk of barotrauma with PVL was 0.90 (95% CI 0.66, 1.24, p = 0.53) (Table 5). We found no interaction between barotrauma effects and within-study gradients in tidal volume or airway pressures (data not shown).

**Table 5:** What is the effect of pressure- and volume-limited ventilation compared to traditional mechanical ventilation with respect to survival and other outcomes in adult ALI and ARDS?

		*Quality Assessment*					*Summary of Findings*			
							Quality†	Relative Risk (95% CI)p-value	Illustrative risks	
Outcome	No. of patients (studies)	Risk of Bias	Inconsistency§	Indirectness¶	Imprecision	Publication Bias			Example control rate	Associated risk with PVL
Hospital mortality	1,749 (10)	Inability to blind.2 trials stopped early with few events and large effects; were also confounded by ‘open lung’ strategies.	p = 0.07I^2^ = 45.6%Varied populations, interventions.Not robust in sensitivity analyses	Direct	Precise	Undetected	Moderate due to (inconsistency)	0.84(0.70 – 1.00) p = 0.05	40%	33.6%(28.0 – 40.0)
Barotrauma	1,497 (7)	Inability to blind.	p = 0.24I^2^ = 25.3%Varied populations, interventions	Direct	Imprecise	Undetected	Moderate due to (imprecision)	0.90(0.66 – 1.24) p = 0.53	NS	NS
Paralysis	1,202 (5)	Inability to blind.	p = 0.004I^2^ = 59%Varied populations, interventions, measurements	Direct	Precise	Not assessed	Moderate due to (inconsistency)	1.37(1.04 – 1.82) p = 0.03	30%	41.1%(31.2 – 54.6)
Dialysis	173 (2)	Inability to blind.	p = 0.26I^2^ = 22.8%Varied populations, interventions	Direct	Imprecise	Not assessed	Moderate due to (imprecision)	1.76(0.79 – 3.90) p = 0.16	NS	NS

**Legend**

* This Summary of Evidence Table corresponds to the GRADE method of summarizing clinical research evidence.

§ Inconsistency is described by the p-value corresponding to the Cochrane Q test for heterogeneity, the I^2^ statistic, and differences among study methods.

¶ Indirectness relates to proximity to the question of survival benefit.

† The quality of randomized trial evidence can be downgraded for risk of bias, inconsistency, indirectness, imprecision, or publication bias.

#### Paralysis

Five trials [38]–[41], [43] (N = 1,202) reported on the use of paralysis. The proportion of patients receiving paralytic agents ranged from 21.7% [40] to 74.1%. [38] Compared to patients receiving traditional ventilation, significantly more patients managed with a PVL approach received paralysis (RR 1.37; 95% CI, 1.04, 1.82; p = 0.03).

#### Dialysis

Two trials [39], [40] including 173 patients reported on study initiation of dialysis. We found no effect of PVL on rates of acute dialysis (RR 1.76 95% CI, 0.79, 3.90, p = 0.16).

Table 5 applies the GRADE approach to summarize the quality of evidence and relative and absolute estimates of effect of PVL for the 4 binary outcomes of this review (mortality, barotrauma, paralysis and dialysis) [19]. Limitations of the evidence include methodologic weaknesses in the studies, confidence intervals that bordered on no effect for mortality and use of paralytic agents, and inconsistent results for mortality. We chose to rate down the quality of evidence for mortality primarily on the basis of inconsistency of results.

#### Evolution of gas exchange and organ system failure

We provide descriptive data related to the evolution of pulmonary and non-pulmonary organ dysfunction. While the evolution of gas exchange was measured variably, between-group differences were modest and inconsistent. Measurements of oxygenation over the first week included PaO_2_ in 4 trials [34], [38], [41], [43], FiO_2_ in 5 trials [38], [40], [41], [43], [45] and PaO_2_/FiO_2_ ratio in 4 trials [38], [39], [43], [45]. Of these, 3 trials [39], [43], [45] noted significant differences in PaO_2_/FiO_2_ with 1 trial favoring the traditional strategy, [43] and 2 trials (using higher PEEP) [39], [45] favoring a PVL approach. One trial [34] noted a significantly higher PaO_2_ favoring the non-experimental arm. While the ARDSNet trial [43] observed lower oxygen requirements among patients treated with a traditional approach, Villar and colleagues [45] found that PVL-treated patients required significantly lower FiO_2_. The latter experimental strategy, however, included higher PEEP levels in the PVL strategy. A significantly higher partial pressure of carbon dioxide (PaCO_2_) (or daily mean PaCO_2_) and significantly lower pH levels (or daily mean pH) over the first week were consistently reported with a PVL strategy in 6 trials [34], [38], [39], [41], [43], [46] and 3 trials [34], [39], [43], respectively.

Three trials [38], [43], [45] evaluated organ system failures. Brochard and colleagues [38] noted a comparable incidence of multiple organ system failure (41%) and nonsignificant differences in the mean number of specific failing organs at day 3, 7 and 14. ARDS Network investigators [43] found significantly fewer days of non-pulmonary organ system failure with PVL compared to traditional ventilation (15±11 vs. 12±11; p = 0.006) including days of circulatory, coagulation or renal failure. Villar et al, [45] found that both groups developed additional organ failures after randomization, with patients in the control group developing significantly more organ system failure than patients in the low tidal volume group (p<0.001).

Data on duration of ventilation, ventilator-free days, length of ICU and hospital stay were infrequently reported or reported non-uniformly, which precluded meta-analysis.

## Discussion

This systematic review of 10 RCTs comparing PVL strategies to ventilation strategies designed to approach more traditional ventilatory goals in ALI and ARDS suggests that PVL reduces mortality. However, this finding was not robust in sensitivity analyses and the confidence intervals include unity, so some uncertainty remains. We did not detect dose-response interactions between treatment effect and the magnitude the differences in tidal volumes or airway pressures. However, control group ventilation strategies did not achieve the full range of traditional tidal volumes; mean tidal volumes were consistently at the lower end of the traditional range. We found no effects of PVL ventilation on barotrauma, which was an anticipated benefit. We observed more acidosis with PVL strategies and a significant increase in the use of paralytic agents.

The analysis in which we pooled survival data from trials involving 1,749 patients may represent an overestimate of treatment effect. The summary estimate suggests a 16.0% reduction in the relative risk of mortality with PVL, and the confidence intervals suggest that the relative risk of mortality might be reduced as much as 30.0%, or not at all. While the ARDS Network trial [43] contributed 19.9% of the weight toward this summary estimate, the trials of Amato [39] and Villar [45] contributed 8.8% and 9.1% weight (total 17.9% weight), respectively. Although 3 trials [39], [43], [45] stopped early for benefit, the ARDS Network trial [43] enrolled 861 patients and contributed a large number of events (>100) in each treatment arm, and its estimate of treatment effect is therefore unlikely to be biased. However, the primary analysis is strongly influenced by 2 small trials that employed additional open lung strategies and stopped early for benefit after only a small number of events (30 hospital deaths in one trial [39], and 42 in the other [45]). One trial [39] used a correction for multiplicity proposed by Peto [52] et al and Geller [53] et al with a significance level of <0.001 while the other trial [45] used a two-step stopping rule when the between group difference in ICU mortality was ≥20% with at least 45 patients in each arm. Treatment effects from these trials are likely too optimistic. While the corrections proposed for multiple sequential analyses may control for type 1 error they cannot prevent associated changes in the magnitude of treatment effect caused by early termination of small trials. [54] This issue persists in the sensitivity analysis that excluded a single randomized trial [37] not reported as such in the manuscript, but stated to be randomized in discussions with the first author. The summary estimate, excluding this trial, suggests a nonsignificant 14.0% reduction in the relative risk of mortality with PVL, with confidence intervals suggesting that the relative risk of mortality might be reduced as much as 28.0% or increased by as much as 4.0%. While the ARDS Network trial [43] contributed 21.4% of the weight toward the summary estimate of effect in this analysis, the trials of Amato [39] and Villar [45] contributed 9.49% and 9.8% weight (total 19.3% weight), respectively.

Whether or not open lung strategies improve survival is a subject of ongoing controversy. A recent meta-analysis of 6 RCTs involving 2,484 patients and comparing 2 different levels of PEEP (with or without other interventions) suggested that the use of high levels of PEEP may have an independent beneficial effect on mortality with an absolute risk reduction of approximately 5%. [55] Meanwhile, empirical evidence shows that early stopping for perceived benefit, particularly after few events, results in inflated estimates of treatment benefits in RCTs and in subsequent meta-analyses. [18] A sensitivity analysis excluding 2 stopped-early trials [39], [45] using PVL and an open lung approach (RR 0.90; 95% CI, 0.74, 1.09; p = 0.27) was inconclusive and could not rule out the possibility of important benefit or harm with PVL. Our results are congruent with the lack of apparent dose-response interactions, the lack of effect on barotrauma, and the inconsistency of study findings with respect to rates of non-pulmonary organ dysfunction.

Historically, investigators using high tidal volumes reported high rates of barotrauma in clinical practice. [56] The highest barotrauma rates in this review were noted in trials that either did not impose pressure constraints or permitted high airway pressures. Recent epidemiologic studies in ALI and ARDS have shown that the incidence of barotrauma is lower than in historical series where tidal volumes were much higher. [57] However, our pooled analysis did not detect a reduction in barotrauma with PVL, nor did we detect an interaction with between-group gradients in airway pressures or tidal volumes.

A notable physiological effect of PVL strategies is respiratory acidosis. Among 6 trials reporting on the evolution of arterial carbon dioxide levels there were significantly higher arterial partial pressures of carbon dioxide and lower pH levels over the first week of study. Analyses exploring a possible interaction between tolerance for acidosis and survival effects of PVL were inconclusive.

The higher rate of paralysis with PVL strategies may be related to higher rates of respiratory acidosis and ventilator dysynchrony. While early observational studies suggested that neuromuscular blockade may increase rates of ICU polyneuropathy, a recent RCT suggested that neuromuscular blockade, itself, may improve gas exchange and biological markers of lung injury. [58] A follow-up trial is presently underway to evaluate the effect of paralytic agents on ARDS mortality (NCT00299650).

Pooling results in a systematic review with meta-analysis implicitly assumes that the trials are sufficiently similar with respect to populations, study interventions, measurement of outcomes and methodologic quality that one could reasonably expect a similar underlying treatment effect. While this was our assumption in pooling data across trials, we launched this review with the explicit goal of testing hypotheses to explain the differences among study results. The most prominent of the 10 trials is the ARDS Network trial [43] which enrolled more patients than all of the other trials combined and stopped early after a relatively large number of events, found a significant mortality reduction with PVL and contributed the largest weight to the pooled estimate of effect for mortality in this review. While this trial galvanized a change in the management of ALI and ARDS, we reviewed it in the context of all available RCT evidence comparing the alternative approaches to ventilator management and pooled it with other trials using conservative methods.

### Strengths and Limitations

This review was strengthened by following a predetermined protocol for review methods and statistical analysis. Our extensive search strategy allowed us to identify an additional 341 patients from 3 trials [37], [42], [46] not included in prior reviews. [13]–[16] We used duplicate, independent citation screening and data abstraction. We corresponded with lead investigators for each trial. In addition to critically appraising usual methodologic quality of randomized trials, we also considered design characteristics specific to this field that might lead to biased estimates of treatment effect, most notably the confounding effects of open-lung ventilation. Finally, based on between-study variation in clinical protocols and statistically significant heterogeneity, we used random effects models which take into consideration both between-study and within-study variation for pooling data across studies. Random effects models typically generate wider confidence intervals than fixed effects models in the presence of appreciable between-study variability in results. [59] Overall, most trials in this review included measures to reduce bias following randomization and were of moderate quality in reporting important outcomes including mortality, barotrauma, paralysis, and dialysis (see Table 5). However, heterogeneity among trials in adopting these measures and in reporting their results may limit interpretation of the pooled results.

### Conclusion

This systematic review suggests that PVL strategies for mechanical ventilation in ALI and ARDS may reduce mortality and, therefore, supports the current practice to ventilate these patients with low tidal volumes. However, we did not find a dose-response effect and this borderline significant finding was not robust in sensitivity analyses. Therefore, some uncertainty regarding the effect of PVL ventilation remains.
